# Supplementary material for: EP4 as a Negative Prognostic Factor in Patients with Vulvar Cancer
Source: Cancers (Basel). 2021 Mar 19;13(6):1410. doi: 10.3390/cancers13061410 (PMC8003514; doi:10.3390/cancers13061410)
Supplement: Supplementary file 1 [file cancers-13-01410-s001.pdf]

cancers

MDPI

Article

EP4 as a Negative Prognostic Factor in Patients with Vulvar Cancer

Anna Buchholz [1], Aurelia Vattai [1], Sophie Fürst [1], Theresa Vilsmaier [1], Christina Kuhn [1,2], Elisa Schmoeckel [3], Doris Mayr [3], Christian Dannecker [1,2], Sven Mahner [1], Udo Jeschke [1,2,*] and Helene H. Heidegger [1]

1 Department of Obstetrics and Gynecology, University Hospital, LMU Munich, Marchioninistraße 15, 81377 Munich, Germany; buchholz.anna@web.de (A.B.); aurelia.vattai@med.uni-muenchen.de (A.V.); sophie.fuerst@med.uni-muenchen.de (S.F.); Theresa.Vilsmaier@med.uni-muenchen.de (T.V.); christina.kuhn@uk-augsburg.de (C.K.); christian.dannecker@med.uni-augsburg.de (C.D.); Sven.Mahner@med.uni-muenchen.de (S.M.); Helene.Heidegger@med.uni-muenchen.de (H.H.H.)
2 Department of Obstetrics and Gynecology, University Hospital Augsburg, Stenglinstrasse 2, 86156 Augsburg, Germany
3 Department of Pathology, LMU Munich, Thalkirchner Str. 142, 80337 Munich, Germany; elisa.schmoeckel@med.uni-muenchen.de (E.S.); doris.mayr@med.uni-muenchen.de (D.M.)
* Correspondence: udo.jeschke@med.uni-muenchen.de; Tel.: +49-89-4400-54240

Citation: Buchholz, A.; Vattai, A.; Fürst, S.; Vilsmaier, T.; Kuhn, C.; Schmoeckel, E.; Mayr, D.; Dannecker, C.; Mahner, S.; Jeschke, U.; et al. EP4 as a Negative Prognostic Factor in Patients with Vulvar Cancer. *Cancers* **2021**, *13*, 1410. https://doi.org/10.3390/cancers13061410

Academic Editor: David Wong

Received: 7 January 2021
Accepted: 17 March 2021
Published: 19 March 2021

Publisher's Note: MDPI stays neutral with regard to jurisdictional claims in published maps and institutional affiliations.

Copyright: © 2021 by the authors. Licensee MDPI, Basel, Switzerland. This article is an open access article distributed under the terms and conditions of the Creative Commons Attribution (CC BY) license (http://creativecommons.org/licenses/by/4.0/).

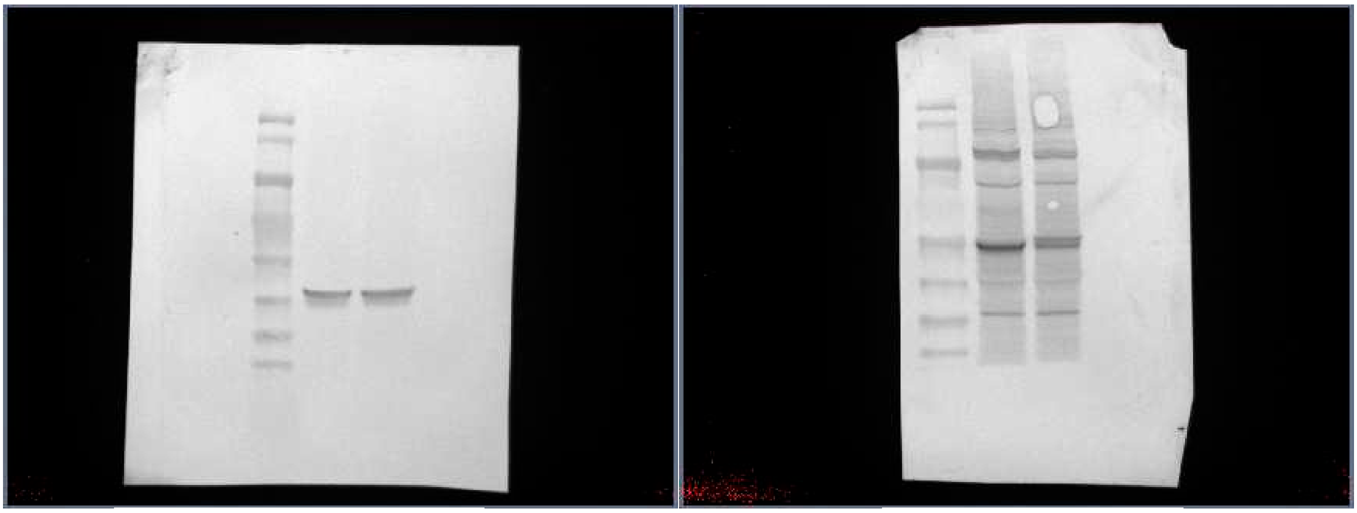

a. Western blot of beta aktin b. Western blot of EP4

Figure S1. uncropped western blots with molecular weight markers.
